# Short-term effects of kinesiology taping on static and dynamic balance in healthy subjects

**DOI:** 10.3389/fnhum.2024.1397881

**Published:** 2024-06-04

**Authors:** Tianyu Zhou, Lin He, Fasen Huang, Tim Sharp, Xiao Hou

**Affiliations:** ^1^School of Healthcare Sciences, Cardiff University, Cardiff, United Kingdom; ^2^Department of Rehabilitation Medicine, West China Hospital, Sichuan University, Chengdu, China; ^3^Shenzhen Traditional Chinese Medicine Hospital, Shenzhen, China; ^4^School of Sport Science, Beijing Sport University, Beijing, China

**Keywords:** immediate effects, kinesiology taping, quadriceps muscles, static balance, dynamic balance

## Abstract

**Background:**

As a therapeutic tool, kinesiology taping (KT) has become increasingly popular for musculoskeletal injuries utilized by physiotherapists. KT has been found to have effects on facilitating muscle strength by generating a concentric pull on the fascia. However, little is known about KT in the improvement of dynamic and static balance. This study aims to explore whether KT on the quadriceps muscle has any immediate effects on static and dynamic balance.

**Methodology:**

Twenty-seven healthy individuals (13 males and 14 females, aged 22 to 29) were recruited in a crossover study with two conditions: KT and no taping. KT was applied to the quadriceps muscle for the taping group, with the control receiving no taping. Pre- and post-test measurements were taken to give an indication of the effect of the tape on balance performance. Center of Pressure Excursion (COPE) and Time to Stabilization (TTS) when landing from a hop test and Y Balance test combined score (YBTCS) were used to assess a stabilizing balance activity and a dynamic balance. The pre- and post-intervention were collected, with differences explored using repeated measures ANOVA with time and condition (tape) factor analysis.

**Results:**

We found a significant improvement (*p* ≤ 0.05) with a moderate to large effect size in YBTCS between KT and no taping, indicating enhanced balance performance in the KT group. However, no significant difference (*p* ≥ 0.05) with small to moderate effect size was found in COPE or TTS between the two conditions during landing tests, suggesting similar balance capabilities in these specific measures.

**Conclusion:**

The use of KT shows no significant immediate effect on static balance in healthy individuals when applied to the quadriceps muscles; however, it demonstrates a positive immediate effect on dynamic balance.

## 1 Introduction

Kinesiology Taping (KT), an elastic therapeutic tape developed in the 1970s by chiropractor Dr. Kenso Kase, is widely used for treating sports injuries and various other disorders (Tran et al., 2023). KT is reportedly different to standard athletic tape in that it provides support but an unrestricted range of movement; this is theorized to allow KT to facilitate muscle function and enhance neuromuscular control (Yin, 2020). KT has become increasingly popular in recent times, becoming increasingly evident in various sports and a diverse range of athletes with various injuries (Bischoff et al., 2018; Nunes et al., 2021). The proposed beneficial effects of KT have led to it becoming part of the daily treatment and rehabilitation programs of many sporting therapists (Dolphin et al., 2021). It is proposed to activate the pain-suppressing system and release the compression on nociceptors to relieve the pain (Montalvo et al., 2014; Deng et al., 2021). Additionally, KT is suggested to enhance lower-extremity muscle activity, strength, and tone, as well as dynamic stability (Kim and Kim, 2021). Studies have also demonstrated significant improvements in gait functions (Biz et al., 2022) and agility performance (Sarvestan et al., 2018). These enhancements have potential implications for the prevention of sports injuries and in reducing the overall incidence of injuries (Williams et al., 2012). This evidence further supports the diverse applications of Kinesio Tape in both therapeutic and athletic settings, underscoring its value as a multifunctional tool in sports medicine and physical therapy.

Despite these proposed physiological benefits, the enhancement of balance induced by KT has not been widely recognized, with a wide range of contradictory studies (Torres et al., 2016; Bischoff et al., 2018; Gholami et al., 2020). Balance is considered a key component of normal daily activities, such as walking, running, and climbing stairs (Lengkana et al., 2020). Proper balance will enable a person to perform activities or movements effectively, and the functional integration of multiple systems in developing excellent balance is fundamental to sporting and functional success (Conner et al., 2019). Balance activities are also crucial to successful performance and injury prevention programs (Pérez-Gómez et al., 2022). Any intervention that can improve balance, therefore, has the potential to improve performance and help prevent injury. However, little is known about KT’s effects on improving balance. Some evidence has been published that has attempted to gain an understanding of KT’s impact on muscle function and improving balance (Kim et al., 2014; Hosp et al., 2017; Zulfikri and Justine, 2017). KT can increase sensory feedback from mechanoreceptors in the skin, muscles, and joint capsules (Hosp et al., 2017). The afferent feedback of mechanoreceptors may assist in maintaining muscle spindle information accuracy, which contributes to improving proprioception (Kase, 2003). Increased proprioception provides more sensory signals to the central nervous system by integrating proprioceptive information, improving joint position sense during static and dynamic conditions (Kim et al., 2014). These mechanisms are thought to improve the efficiency of the neuromuscular system and minimize postural sway, thereby improving the ability to maintain balance efficiently during movement (Zulfikri and Justine, 2017).

Two distinct circumstances are associated with different postural control dynamics and strategies: (1) static balance and (2) dynamic balance. Static balance is the body’s ability to remain relatively stable, with the most minor movement when standing on one leg (Lengkana et al., 2020). Dynamic balance refers to the body’s capacity to maintain its position and stability in response to perturbations (Lengkana et al., 2020). Given that the evidence on the effect of KT in balance outcomes is currently lacking, this study aims to explore whether KT has any immediate effects on the quadriceps muscles for improving static and dynamic balance compared with the control condition of no taping in healthy subjects during landing from a hop and Y Balance test.

## 2 Materials and methods

### 2.1 Study design

The study was a prospective, randomized controlled crossover trial designed to evaluate the effectiveness of KT on static and dynamic balance. The crossover design enhances precision, as participants serve as their own controls, thereby increasing the study’s statistical power (Robinson et al., 2019). This research was approved by the Cardiff University of School of Healthcare Sciences ethics committee in accordance with the Helsinki Declaration.

### 2.2 Participants

This study recruited 27 physically active, healthy young adults (13 males and 14 females), aged between 22 and 29 years, from Cardiff University. Exclusion criteria included: (1) recent lower limb injuries affecting function or causing pain; (2) a recent history of balance disorders, neurological disorders, ear infections, or visual disturbance; (3) the presence of open wounds, injuries, or infection/ irritations of the skin around the knee; (4) a history of allergy to tape; (5) pregnancy or breastfeeding. Prior to the study, all participants read and signed an informed consent form.

### 2.3 Randomization and blinding

Participants were randomly allocated to either the KT or control groups using sealed envelopes to ensure randomness in the group assignment. The allocation was counterbalanced, ensuring that an equal number of participants performed each test. All researchers involved were kept blind to the group allocations and the randomization sequence to prevent bias. Due to the nature of the intervention, where the KT group received KT on the dominant leg’s quadriceps muscle and the control group did not receive any tape, complete blinding of participants was not feasible. However, to maintain blinding of the outcome assessors, participants wore long trousers during the data collection phase to conceal whether KT was used.

### 2.4 Pre-setting procedures

Before the experiment, three specific functional tests were conducted to determine the leg dominance of each participant: the ball-kick test, the step-up test, and the balance recovery test (Paillard and Noé, 2020). In the ball kick test, the leg used to kick a ball is considered dominant. In the step-up test, the leg used to step on a bench is dominant. In the balance recovery test, the dominant leg is identified as the leg used to step out for balance recovery after a push from behind. Among these three specific functional tests, the limb used for at least two functional tests was categorised as the dominant limb. Once identified, this limb was targeted for KT application. Subsequently, all participants performed a supervised five-minute warm-up using a cycle ergometer, with each individual choosing their own speed (Wattbike, United Kingdom, model: Pro Indoor Cycle).

### 2.5 KT application

**Figure 1:**
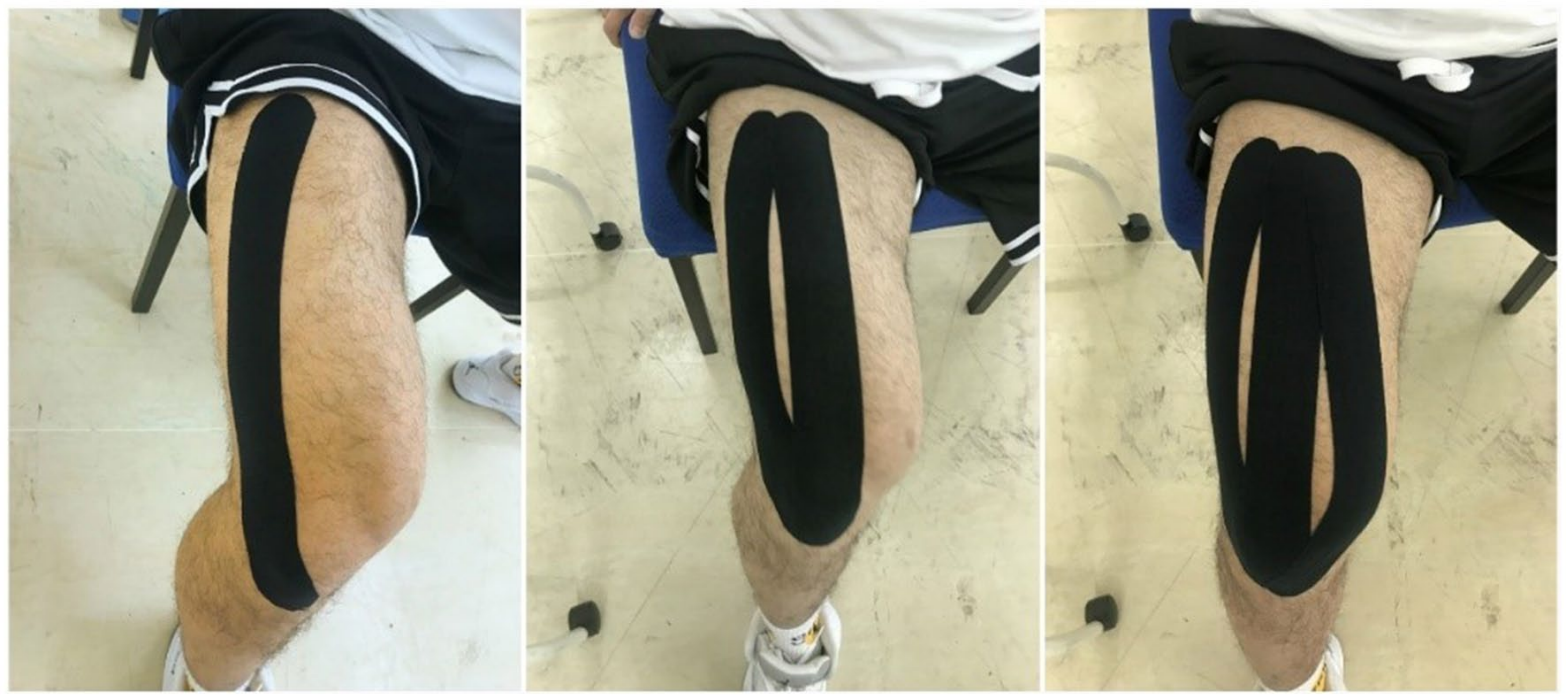
Kinesiology tape applied on the quadriceps muscle.

Prior to the placement of the KT, the anterior thigh was shaved and cleaned with alcohol. KT (SpiderTech, Canada) was applied using the technique proposed by Kase (2003). Participants were applied with KT in a relaxed position with the knee flexed 90° to stretch the quadriceps muscles. Three strips of ‘I’ tape were applied on the three quadriceps muscles of the dominant leg from the muscle origin to its insertion. The first strip of tape was applied from a point 10 cm below the anterior inferior iliac spine to the lateral inferior aspect of the patella for the vastus lateralis muscle. Another strip of tape was applied from a point 10 cm below the anterior inferior iliac spine to the inferior border of the patella for the rectus femoris muscle. The last strip of tape was applied from a point 10 cm below the anterior inferior iliac spine to the medial inferior aspect of the patella for the vastus medialis muscle (Figure 1). This taping technique, which is assumed to increase the number of motor units recruited, provides mechanical stability to the knee joint (Kase, 2003).

The tape color was black, and the stretching tension was 80% (Khalili et al., 2022). To ensure the tension of KT is as close as possible to 80%, the distance between the application points (origin and insertion) was measured for the quadriceps of each participant. To determine the length of tape to be cut, the following calculations were made: Final length of KT = the distances between the application points minus 6 cm (which is the length of two anchor points with no tension) and divided by 1.8 (meaning that 80% tension is applied). The same certified KT practitioner performed each tape application to ensure consistency throughout the study.

### 2.6 Landing from a hop test

**Figure 2:**
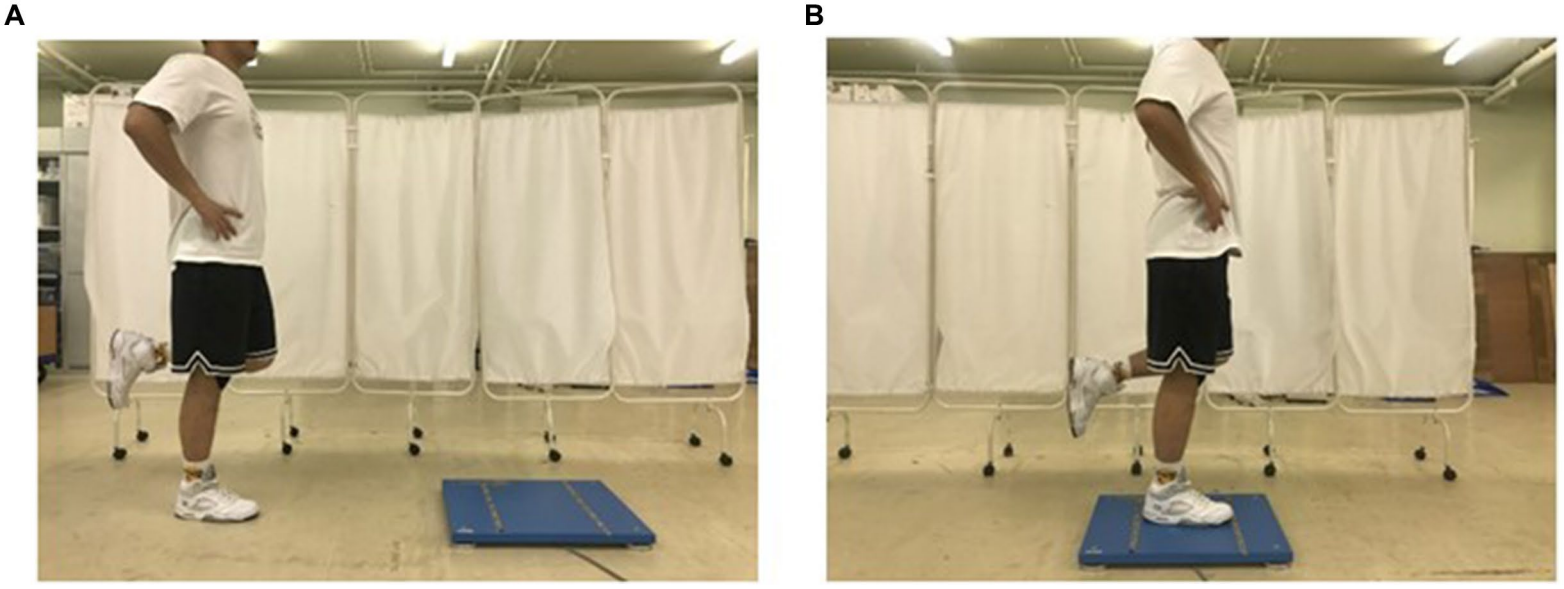
The single-leg landing test **(A)** from the floor toward the center of a force platform; **(B)** standing on the tested lower limb to stabilization.

The test involved balancing on a force platform (Kistler, United Kingdom, model: 9260AA) with one foot after a hop in the anterior direction (Riemann and Schmitz, 2012). Prior to the test, the maximum single-leg hop distance was measured by having participants perform three maximum effort hops on the dominant leg, with the longest distance from these trials recorded as the maximum hop distance. The average of these maximum distances was then used to calculate 80% of the maximum hop distance for each participant. Participants were instructed to balance on their dominant leg in front of the force platform, equivalent to 80% of the maximum single-leg hop distance. The opposite leg of the participants was held with the knee flexed to 90° and the tibia parallel to the floor. When the signal sounded, the participants were asked to hop forward to the central area of the force platform and remain stable with one leg for 30 s (Figure 2). The force platform automatically recorded the time for 30 s. During the test, participants were asked to keep their hands on their iliac crests, described as an akimbo position (Trojian and Mckeag, 2006). In addition, participants were verbally instructed to concentrate on the hop and standing on the force platform (Gerbino et al., 2007). The test should be repeated if the force plate is touched with the non-weight-bearing leg or the hand is away from the waist when landing (Huang et al., 2014). Participants were familiarized with the equipment through practice trials prior to data collection.

### 2.7 Y balance test

**Figure 3:**
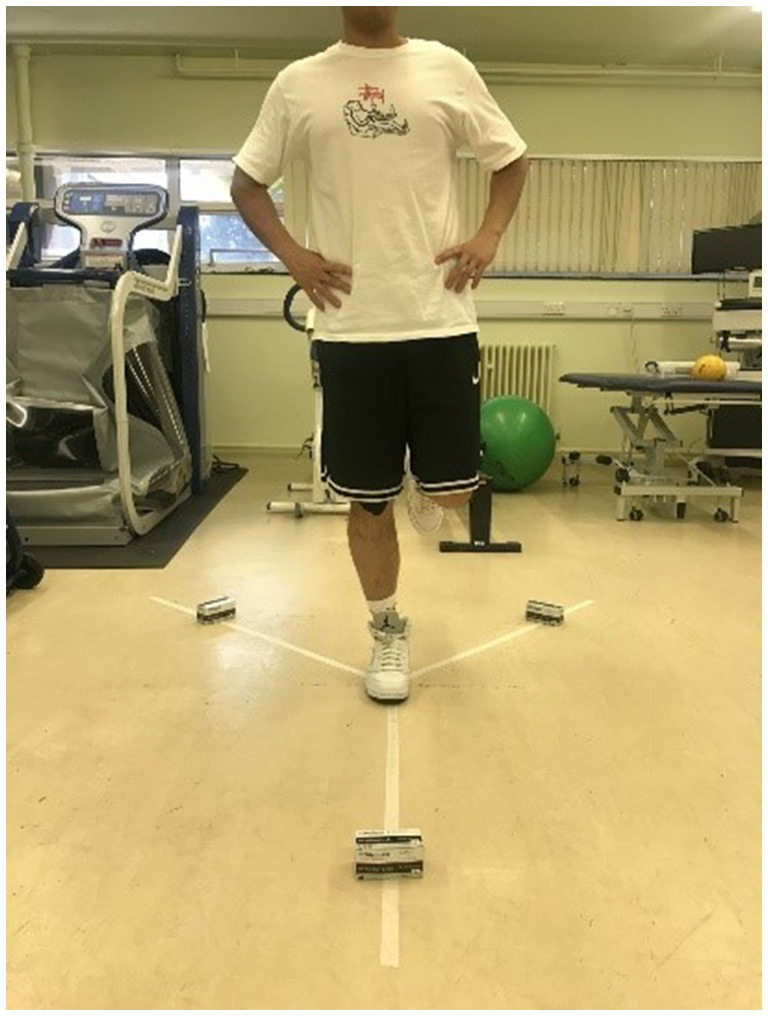
The starting position of the Y-balance test.

**Figure 4:**
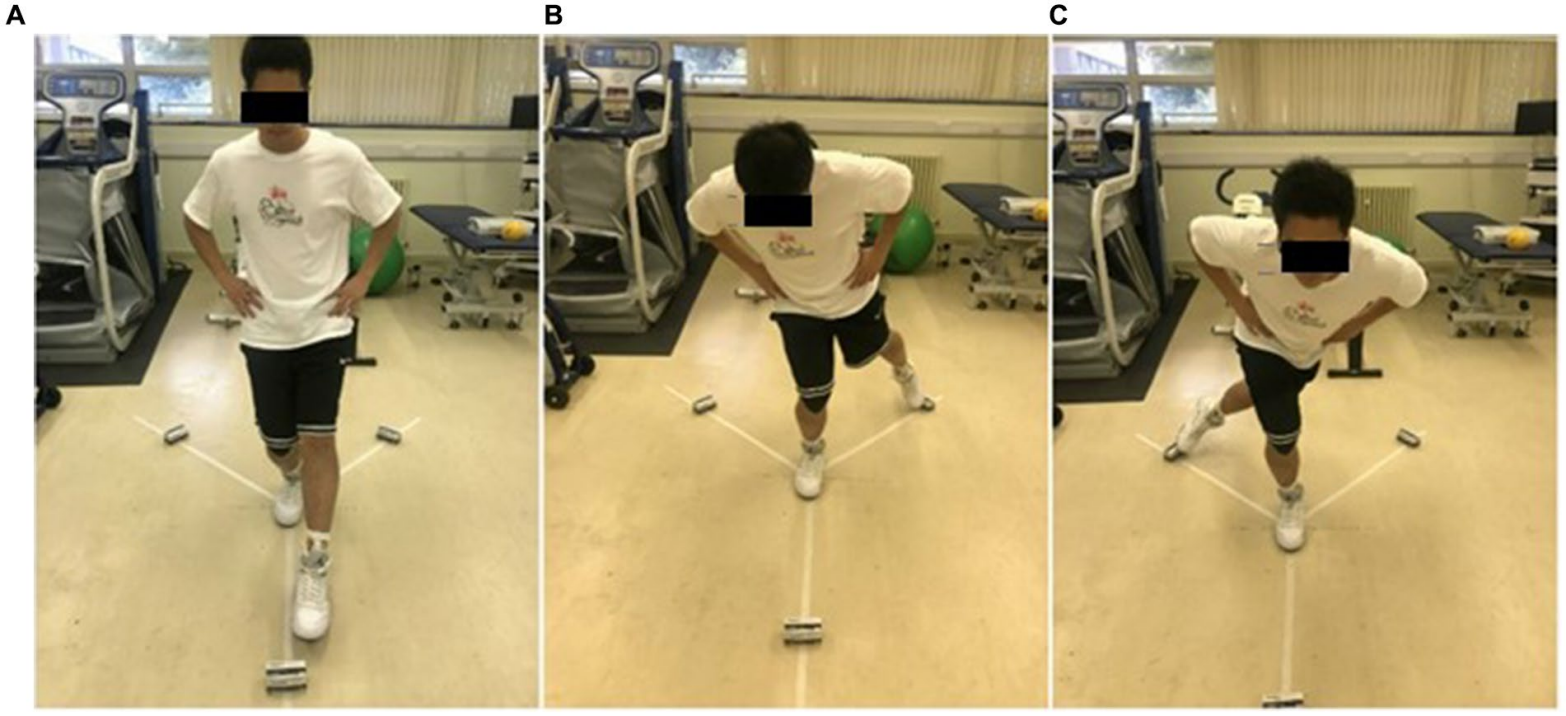
Y balance test directions. **(A)** Anterior; **(B)** Posteromedial; **(C)** Posterolateral.

The Y Balance Test (YBT) involved participants standing on one leg and extending the other leg as far as they could in three distinct directions: anterior, posteromedial, and posterolateral (Powden et al., 2019). The posterior directions are each positioned 135 degrees from the previous and 90 degrees between them (Neves et al., 2017). All participants were allowed to engage in practice trials to acquaint themselves with the task. Leg lengths were measured bilaterally in the supine position, extending from the inferior border of the anterior-superior iliac spine to the inferior border of the medial malleolus (Desai et al., 2013). Participants were instructed to stand stably at the center of three lines, balancing on the dominant leg to which the KT was applied while placing their hands on their hips (Figure 3). Subsequently, participants were asked to push the first box as far forward as possible with their raised foot before returning to the upright position. The anterior test was performed first, followed by the posteromedial and posterolateral tests (Figure 4). The maximum reach distance of each trial was marked and recorded, which was used to calculate the YBT combined score. During the test, the participants were instructed to avoid touching the floor with their non-dominant feet until returning to the starting position so that it did not provide support in maintaining balance. If the individual cannot perform the test according to the above criteria in six attempts, he fails in the direction (Plisky et al., 2009). The dominant foot must maintain contact with the box until the end of the reach, and the boxes cannot be flicked or kicked to improve the result.

### 2.8 Outcome measurement

The center of pressure excursion (COPE) derived from landing from a hop test was used to evaluate static balance. COPE measures deviation in the location of the center of pressure on the supporting surface, which has been recognized as a reliable measure of postural steadiness during standing (Li et al., 2016). Dynamic balance was quantified by the time to stabilization (TTS) collected from landing from a hop test and Y-balance Test Combined Score (YBTCS) collected from the Y-Balance Test (YBT). TTS can reflect when the range of variation of ground reaction force returns to baseline variation when the body transitions from a dynamic to a static state, which has been verified as a reliable and precise measure of dynamic postural stability (Kremenic et al., 2009; Ringhof and Stein, 2018). YBT has also been considered one of the most prominent tools in the literature for assessing the dynamic balance of the lower extremities (Powden et al., 2019). Given the crossover design of the study, each participant underwent two testing sessions on the same day, separated by a 90-min washout period to mitigate any carry-over effects (Robinson et al., 2019). All participants were required to wear standardized shoes during data collection to ensure consistency across tests and reduce any potential variability caused by different footwear.

### 2.9 Data processing

Data for COPE were calculated and collected using BioWare Software (Version 4.0.x) on the lab computer, while data for TTS were calculated and collected using MATLAB software (Version 2018). The maximum reach distances for three directions during the Y-Balance test were collected using a Laser Distance Meter (RSCLLDM-50H, United Kingdom). The YBTCS was then calculated and collected as the sum of maximum reach distances in three directions divided by 3 times limb length and then multiplied by 100% (Kim et al., 2022).


$$\begin{array}{l} \mathrm{Overall}\;\mathrm{composite}\;\mathrm{score}:\\ \frac{\mathrm{Anterior}+\mathrm{Posteromedial}+\mathrm{Posterolateral}}{3\times \mathrm{Lower}\;\mathrm{Limb}\;\mathrm{Length}}\times 100 \end{array}$$

### 2.10 Statistical analysis

To test the effect of the tape on the ability within the three balance outcome measures, a repeated measures ANOVA was conducted on the pre and post-test scores for both conditions (tape and control (no tape)). Considering the effect size of 0.5, α of 0.05, and a power of 80%, a minimum paired sample size of 27 participants was necessary to detect a difference between the two conditions for this study. The Statistical Package SPSS version 25 was utilised for all statistical analyses. Statistical significance was set at an alpha level of less than 0.05.

## 3 Results

A total of 27 healthy adults (13 males and 14 females) who met the inclusion and exclusion criteria participated in the study. All subjects completed all two testing sessions on the same day without anyone withdrawing. Only one subject (1 female) felt slightly uncomfortable after completing the tests. However, the discomfort disappeared within 12 h. The demographic data of the subject’s age, height, and weight, including means and standard deviations, is presented in Table 1.

**Table 1:** Mean (standard deviation) of age, height, and weight of subjects.

	Males (*n* = 13)	Females (*n* = 14)	Total (*n* = 27)
Age(yrs.)	25 (2)	26 (5)	25 (4)
Height(cm)	176 (6)	164 (6)	170 (8)
Weight(kg)	73.6 (9.1)	58.8 (13.2)	65.9 (13.6)

The YBTCS within the taping group showed an increase from 1.02 in the pre-test to 1.05 in the post-test, whereas the control group remained nearly unchanged (pre-test 1.05, post-test 1.04) (see Table 2). Statistical analysis revealed a significant improvement in YBTCS for the condition (*F* = 7.60, *p* = 0.01), while no significant changes were observed for time (*F* = 0.55, *p* = 0.47) (see Table 3). For the COPE, both the taping and control groups demonstrated similar mean values from pre-test to post-test, with the KT group changing from 0.06 m (SD = 0.05) to 0.07 m (SD = 0.06) and the control group from 0.07 m (SD = 0.05) to 0.06 m (SD = 0.04) (see Table 2), with no statistically significant differences found either by condition (*F* = 0.00, *p* = 0.99) or over time (*F* = 0.01, *p* = 0.94) (see Table 3). Similarly, the TTS showed no significant changes, with the taping group changing from a pre-test mean of 2.65 s (SD = 1.74) to a post-test mean of 2.34 s (SD = 1.11), and the control group from 2.38 s (SD = 1.15) to 2.37 s (SD = 1.01) (see Table 2), with no significant effects due to condition (*F* = 0.34, *p* = 0.57) or time (*F* = 0.27, *p* = 0.61) (see Table 3).

**Table 2:** Mean (standard deviation) of the pre- and post-test results for each of the groups and tests.

Tests	Taping		Control (No taping)	
	Pre-test	Post-test	Pre-test	Post-test
COPE(m)	0.06 (0.05)	0.07 (0.06)	0.07 (0.05)	0.06 (0.04)
TTS(s)	2.65 (1.74)	2.34 (1.11)	2.38 (1.15)	2.37 (1.01)
YBTCS	1.02 (0.07)	1.05 (0.07)	1.05 (0.07)	1.04 (0.07)

COPE, Center of Pressure Excursion; TTS, Time to stabilisation; YBTCS, Y Balance Test Combined Score.

**Table 3:** Table showing the significant values among the *F*-test (repeated measures ANOVA).

	Time	Condition	Condition time interaction
COPE(m)	*F* = 0.00, *p* = 0.97, η^2^ = 0.00	*F* = 0.02, *p* = 0.90, η^2^ = 0.00	*F* = 1.17, *p* = 0.29, η^2^ = 0.043
TTS(s)	*F* = 0.27, *p* = 0.61, η^2^ = 0.01	*F* = 0.34, *p* = 0.57, η^2^ = 0.01	*F* = 0.83, *p* = 0.37, η^2^ = 0.03
YBTCS	*F* = 0.55, *p* = 0.47, η^2^ = 0.02	*F* = 7.60, *p* = 0.01*, η^2^ = 0.23	*F* = 9.40, *p* = 0.01*, η^2^ = 0.27

COPE, Center of Pressure Excursion; TTS, Time to Stabilisation; YBTCS, Y Balance Test Combined Score; Condition, Effect of Taping vs. Control (No Taping); Time, Effect of Time (Pre and Post); Condition Time Interaction, Interaction Effect between Condition and Time. ^*^ indicates that the result was significant.

## 4 Discussion

Our results predominantly indicate that the application of KT to the quadriceps muscle significantly enhances dynamic balance as measured by the YBTCS, displaying both statistical significance and a moderate to large effect size. This underscores the substantial practical impact of KT on dynamic balance. Conversely, KT did not show statistical significance in improving dynamic balance according to the TTS and static balance assessed by the COPE. However, the effect sizes for TTS and COPE were small to moderate, suggesting that KT may still have some practical influence on balance despite the lack of statistical significance. These findings highlight the multifaceted role of KT and its varied influence on different aspects of balance.

Although statistically significant, the observed change in the YBTCS of 0.03 m, representing a 1 cm change in each movement of three directions, raises questions about clinical significance. The limited improvement in YBTCS, coupled with the absence of statistically significant differences in TTS and COPE within this study, might potentially be attributable to the sample comprising healthy participants. Hosp et al. proposed the hypothesis that individuals with already “normal” balance abilities may not derive noticeable benefits from external proprioceptive aids, whereas those with impaired proprioception could be more responsive to such interventions (Hosp et al., 2015). This implies that KT might primarily benefit those with poor proprioceptive capabilities or sports injuries. The study by de Almeida Lins et al. (2013) aligns with our findings, as it also observed no significant effect of KT on neuromuscular balance performance among healthy individuals (De Almeida Lins et al., 2013). Another study also found that KT had no effect on knee proprioception in healthy subjects (Torres et al., 2016). Conversely, most research indicates a positive influence of KT on proprioception in patients with traumatic injuries (Williams et al., 2012; Morris et al., 2013; Simon et al., 2014). The findings from another study seem to reinforce this observation, demonstrating that athletes with chronic ankle instability have demonstrated KT’s effectiveness in reducing postural sway during movements and offers a moderate stabilizing effect on the ankles (Biz et al., 2022). These findings highlight the necessity for future research to explore the differential effects of KT between healthy individuals and those with injuries.

The duration for KT remains on the skin may also be a potential reason for its effectiveness. In the current study, the post-test for assessing balance was conducted immediately following the application of KT. Jackson and colleagues found that maintaining KT on the targeted muscle for 48 h could improve static balance, and these improvements persisted for 72 h after removing the tape (Jackson et al., 2016). According to the underlying mechanisms of KT, it might activate skin mechanoreceptors, potentially enhancing muscle spindle reflexes and increasing motor unit excitability over time (Yeung and Yeung, 2016). The body adjusts to the continuous sensory feedback provided by the stimulus, maintaining some effects even after the stimulus has been removed (Simon et al., 2014). Another study assessing the impact of the KT technique on balance in patients with chronic ankle instability found that applying KT for 48–72 h significantly improved balance deficits in these patients (Tetuan, 2014). Consequently, extended KT applications might enhance muscle activation capabilities more effectively than shorter applications, potentially improving balance. However, these findings should be interpreted with caution as insufficient research examining the physiological mechanisms, and further research is thus warranted to fully understand the tape’s long-term effects on balance and muscle activity.

It is also possible that COPE was affected by the KT of single target muscle and measurement tools. In the current study, the static balance was assessed by COPE during the landing from a hop test, which required subjects to land steadily on the force platform and stand with one leg for 30 s. This mainly reflects the whole lower limb’s static balance, such as the hip and ankle joints, rather than only the knee joint. It is hard to detect the apparent change of COPE for static balance when KT was applied only to the knee joints of the participants. The findings of the current study that KT has no immediate effect on static balance are in contrast with the findings of others (Bischoff et al., 2018). Bischoff and his colleagues focused on knee proprioception through the assessment of joint position sense (JPS), which enables accurate assessment of the relative localisation and movement of a particular joint in space, as well as enabling measurement of tension, effort, and balance. Thus, the improvement in static balance shown in the study of Bischoff et al. (2018) was more easily detected than in the current study. The results of another study by Torres and his colleagues reinforce this theory (Torres et al., 2016). They investigated the effect of KT on knee proprioception by assessing JPS and threshold to detect passive movement (TTDPM) in healthy participants. They found that KT positively impacts knee proprioception, although there was only marginal improvement in JPS.

The main limitation of the present study was the use of healthy individuals because participants with poor balance are more likely to benefit from KT than healthy participants. Another limitation may be that no placebo group exists in the current study. The intervention of KT without tension as the placebo group can exclude the effect of KT on cutaneous stimulation. The washout period of only 90 min may be another limitation. The residual effects of KT and remembering and learning from tests influence the results due to the short washout period used in the current study. Another potential limitation of this study is the small sample size. Future research should involve a larger cohort of participants to validate and enhance the generalizability of our findings.

## 5 Conclusion

This study investigated the immediate effects of KT on static and dynamic balance in healthy individuals. While KT demonstrated a significant improvement in dynamic balance as measured by the YBTCS, it did not significantly impact static balance, as assessed through the COPE and TTS measures. These results suggest that KT may enhance dynamic balance capabilities in a healthy population, although its effects on static balance appear limited under the conditions tested. The significant findings related to dynamic balance highlight KT’s potential utility in sports and rehabilitation settings where dynamic movement plays a critical role. However, further investigation is necessary, particularly involving a broader range of participants, including those with balance disorders, to explore the benefits of KT more comprehensively.

## Data availability statement

The original contributions presented in the study are included in the article/Supplementary material, further inquiries can be directed to the corresponding authors.

## Ethics statement

The studies involving humans were approved by Cardiff University of School of Healthcare Sciences ethics committee. The studies were conducted in accordance with the local legislation and institutional requirements. The participants provided their written informed consent to participate in this study.

## Author contributions

TZ: Conceptualization, Investigation, Methodology, Software, Visualization, Writing – original draft, Writing – review & editing. LH: Data curation, Writing – original draft. FH: Formal analysis, Writing – original draft. TS: Project administration, Supervision, Writing – review & editing. XH: Resources, Supervision, Writing – review & editing.
