# Supplementary material for: Short-term effects of kinesiology taping on static and dynamic balance in healthy subjects
Source: Front Hum Neurosci. 2024 Jun 4;18:1397881. doi: 10.3389/fnhum.2024.1397881 (PMC11183502; doi:10.3389/fnhum.2024.1397881)
Supplement: Supplementary file 1 [file Data_Sheet_1.DOC]

**Research Equipment**

Experimental protocol

• Bicycle ergometer (Wattbike, United Kingdom, model: Pro Indoor Cycle)

• Digital weighing scales (Seca, United Kingdom, model: 9081 SV3R))

• Portable height measurer (Leicester, United Kingdom, model: HM-250P)

• Laser distance meter (United Kingdom, RSLLDM-50H)

• Kinesiology tape (SpiderTech, Canada)

• Portable force platform (Kistler, United Kingdom, model: 9260AA)

• Stool (Argos, United Kingdom, model: 6003575)

Data Processing

• Excel (Microsoft, United States, version: 15.12.3)

• BioWare Software (BioWare, United Kingdom, Version 4.0.x, type: 2812A)

• MATLAB software (Mathlab, United States, Version 2018)

• SPSS Statistics (IBM, United States, version: 23.0.0.2)
