# Supplementary material for: Short-term effects of kinesiology taping on static and dynamic balance in healthy subjects
Source: Front Hum Neurosci. 2024 Jun 4;18:1397881. doi: 10.3389/fnhum.2024.1397881 (PMC11183502; doi:10.3389/fnhum.2024.1397881)
Supplement: Supplementary file 2 [file Data_Sheet_2.DOC]

**Raw data for SPSS**

| **Participant** | **Age(yrs.)** | **Height(cm)** | **Weight(kg)** | **COPE** | | | | **TTS** | | | | **YBTCS** | | | |
| --- | --- | --- | --- | --- | --- | --- | --- | --- | --- | --- | --- | --- | --- | --- | --- |
|  |  |  |  | **KT-pre** | **KT-post** | **No Taping-pre** | **No Taping -post** | **KT-pre** | **KT-post** | **No Taping -pre** | **No Taping -post** | **KT-pre** | **KT-post** | **No Taping -pre** | **No Taping -post** |
| **1** | 28 | 177 | 88.8 | 0.142 | 0.132 | 0.205 | 0.121 | 0.770 | 2.345 | 0.320 | 2.925 | 1.037 | 1.011 | 1.087 | 1.115 |
| **2** | 24 | 155 | 50 | 0.040 | 0.052 | 0.059 | 0.060 | 0.935 | 1.785 | 2.280 | 3.670 | 0.935 | 1.055 | 1.076 | 1.069 |
| **3** | 26 | 168 | 72 | 0.148 | 0.125 | 0.101 | 0.098 | 4.485 | 3.870 | 3.780 | 2.670 | 0.972 | 0.985 | 0.961 | 0.957 |
| **4** | 25 | 177 | 65 | 0.078 | 0.058 | 0.074 | 0.051 | 3.915 | 2.780 | 2.630 | 2.590 | 0.988 | 1.035 | 1.053 | 1.043 |
| **5** | 24 | 166 | 65 | 0.034 | 0.200 | 0.170 | 0.171 | 6.100 | 5.410 | 3.560 | 2.660 | 0.926 | 0.949 | 0.923 | 0.896 |
| **6** | 25 | 164 | 98 | 0.059 | 0.036 | 0.095 | 0.016 | 6.415 | 1.440 | 2.420 | 1.690 | 1.051 | 1.089 | 1.088 | 1.051 |
| **7** | 28 | 175 | 84 | 0.087 | 0.115 | 0.071 | 0.075 | 3.825 | 1.320 | 1.610 | 1.060 | 1.045 | 1.072 | 1.012 | 1.039 |
| **8** | 24 | 162 | 64 | 0.038 | 0.012 | 0.002 | 0.023 | 0.980 | 1.670 | 2.035 | 2.760 | 0.976 | 0.995 | 0.971 | 0.986 |
| **9** | 23 | 185 | 75 | 0.014 | 0.023 | 0.013 | 0.013 | 1.085 | 2.560 | 3.450 | 3.460 | 0.898 | 0.968 | 1.014 | 0.886 |
| **10** | 23 | 175 | 60 | 0.035 | 0.020 | 0.046 | 0.073 | 1.665 | 3.095 | 1.205 | 2.210 | 1.100 | 1.131 | 1.154 | 1.093 |
| **11** | 19 | 178 | 77 | 0.052 | 0.052 | 0.083 | 0.014 | 1.995 | 1.580 | 4.005 | 1.440 | 1.025 | 1.071 | 1.134 | 1.065 |
| **12** | 24 | 155 | 46 | 0.093 | 0.035 | 0.011 | 0.013 | 2.445 | 1.045 | 2.825 | 2.820 | 1.114 | 1.149 | 1.137 | 1.111 |
| **13** | 25 | 176 | 77 | 0.051 | 0.043 | 0.065 | 0.014 | 2.415 | 3.435 | 1.945 | 1.825 | 1.046 | 1.066 | 1.032 | 1.080 |
| **14** | 24 | 180 | 73 | 0.110 | 0.105 | 0.131 | 0.101 | 5.290 | 2.330 | 3.415 | 1.520 | 1.113 | 1.168 | 1.042 | 1.081 |
| **15** | 27 | 172 | 65 | 0.033 | 0.004 | 0.010 | 0.031 | 4.625 | 3.310 | 1.545 | 4.285 | 1.145 | 1.152 | 1.104 | 1.129 |
| **16** | 26 | 168 | 60 | 0.126 | 0.168 | 0.074 | 0.077 | 1.345 | 2.320 | 1.200 | 3.720 | 1.044 | 1.126 | 1.119 | 1.131 |
| **17** | 24 | 174 | 63 | 0.057 | 0.071 | 0.091 | 0.095 | 2.325 | 1.980 | 4.585 | 2.510 | 1.067 | 1.093 | 1.031 | 1.057 |
| **18** | 23 | 171 | 52 | 0.013 | 0.041 | 0.018 | 0.019 | 1.060 | 3.215 | 1.060 | 4.920 | 1.055 | 1.058 | 1.102 | 1.085 |
| **19** | 20 | 160 | 47 | 0.030 | 0.043 | 0.032 | 0.059 | 2.595 | 1.425 | 3.260 | 2.120 | 1.072 | 1.105 | 1.072 | 1.036 |
| **20** | 25 | 175 | 70 | 0.122 | 0.153 | 0.082 | 0.084 | 1.435 | 2.055 | 1.365 | 0.775 | 0.935 | 1.033 | 0.966 | 0.974 |
| **21** | 24 | 178 | 71 | 0.017 | 0.036 | 0.044 | 0.021 | 0.375 | 0.835 | 1.250 | 1.395 | 0.943 | 0.926 | 0.977 | 1.012 |
| **22** | 25 | 166 | 52 | 0.056 | 0.138 | 0.071 | 0.132 | 5.830 | 4.495 | 5.025 | 0.970 | 1.045 | 1.019 | 1.097 | 1.086 |
| **23** | 29 | 160 | 54 | 0.008 | 0.011 | 0.034 | 0.055 | 1.215 | 0.795 | 2.410 | 2.395 | 1.039 | 1.135 | 1.148 | 1.084 |
| **24** | 23 | 161 | 53 | 0.140 | 0.104 | 0.056 | 0.130 | 2.485 | 1.855 | 2.220 | 2.430 | 1.078 | 1.031 | 1.010 | 1.018 |
| **25** | 22 | 184 | 89 | 0.028 | 0.076 | 0.191 | 0.061 | 2.100 | 2.395 | 1.960 | 2.140 | 1.042 | 1.037 | 1.032 | 1.054 |
| **26** | 26 | 162 | 48 | 0.053 | 0.028 | 0.040 | 0.083 | 2.595 | 0.915 | 1.350 | 2.185 | 0.892 | 0.902 | 0.867 | 0.893 |
| **27** | 25 | 163 | 61 | 0.034 | 0.017 | 0.002 | 0.023 | 1.340 | 2.990 | 1.550 | 0.975 | 0.976 | 1.063 | 1.043 | 1.004 |
